# To what extent is dot comparison an appropriate measure of approximate number system?

**DOI:** 10.3389/fpsyg.2022.1065600

**Published:** 2023-01-10

**Authors:** Cristina Rodríguez, Roberto A. Ferreira

**Affiliations:** ^1^Millennium Nucleus for the Science of Learning (MiNSoL), Talca, Chile; ^2^Facultad de Ciencias de la Educación, Universidad Católica del Maule, Talca, Chile

**Keywords:** dot comparison, approximate number system, inhibition, visual cues, continuous magnitudes, early childhood, number sense

## Abstract

**Introduction:**

Number sense has been systematically measured using dot comparison tasks. However, recent studies have reported that performance on dot comparison might be influenced inhibitory control and visual properties of dot arrays. In the present study, we analysed the influence of continuous magnitude, inhibitory control, and numerical ratio on the dot comparison performance of preschool children.

**Methods:**

Participants were 517 preschool children from 13 different schools in Chile. Children completed a dot comparison and two inhibitory control tasks. Gebuis and Reynvoet method was used to create well-controlled dot arrays for use in the dot comparison task. A logistic mixed effects model was conducted to predict participants’ dot comparison accuracy. Continuous magnitude and ratio were entered as level-1 predictors and inhibitory control as level-2 predictors.

**Results:**

The results showed that all predictors made a significant contribution to dot comparison accuracy. Furthermore, a significant double interaction (inhibitory control x continuous magnitude) and a triple interaction (inhibitory control x continuous magnitude x ratio) showed that the contribution of inhibitory control skills in dot comparison accuracy depends on the continuous properties of dot arrays and ratio.

**Discussion:**

These findings suggest that preschool children rely more on continuous magnitudes than numerosity in dot comparison tasks. They also indicate that the greater children’s inhibitory control, the more able they are to respond based on numerosity in fully incongruent trials, particularly when ratio is low (easiest items). Taken together, the above findings support the competing processes account provided that both ANS and inhibitory control skills influence performance on dot comparison tasks.

## 1. Introduction

Mathematics is crucial for our everyday lives, from calculating the discount of a product to organising how much time we will dedicate to carrying out tasks to prepare for a meeting, exam, or any other important event. But mathematics crosses the limits of its domain (Duncan et al., 2007). Evidence shows that mathematics skills predict reading (e.g., ten Braak et al., 2022), science (e.g., Korpershoek et al., 2011), and other domain-general and socio-emotional skills (e.g., Romano et al., 2010). Furthermore, mathematical abilities have long-term repercussions on professional development, with individuals with poor mathematical skills presenting higher overall unemployment (Parsons and Bynner, 1997; Reyna and Brainerd, 2007) but higher employment in underpaid manual occupations. Thus, learning mathematics is a crucial goal for countries, schools, and individuals.

Mathematics learning begins long before formal instruction. The first mathematical knowledge is developed very early in infancy (Izard et al., 2009; Libertus and Brannon, 2009). Particularly, the ability to estimate numerical magnitude information is believed to be the foundational skill of mathematical learning and depends on an innate preverbal system, the Approximate Number System (ANS; Feigenson et al., 2004). Many studies have shown that individual differences in ANS are associated with individual differences in mathematical performance (e.g., Price et al., 2007; Mussolin et al., 2010; Mazzocco et al., 2011a). However, some have not found this concurrent relationship between ANS and mathematics (e.g., Iuculano et al., 2008; Holloway and Ansari, 2009; Vanbinst et al., 2012). The scarce evidence of prospective longitudinal research is also mixed; some studies have shown that ANS acuity measured early in infancy predicts later math abilities (e.g., Mazzocco et al., 2011b; Desoete et al., 2012; Libertus et al., 2013; Starr et al., 2013), while others have not (Kolkman et al., 2013; Praet et al., 2013). Additionally, recent meta-analyses showing a significant relation between ANS and math (Chen and Li, 2014; Fazio et al., 2014; Schneider et al., 2017) have reported small overall effects that vary substantially between studies. This phenomenon can be attributed to lack of power (Chen and Li, 2014) and differences in ANS tasks, some of which are not even reliable (Clayton et al., 2015, 2019).

In the last few years, a growing number of studies have been focused on the validity of the method to measure ANS (e.g., Gebuis and Reynvoet, 2011; Clayton et al., 2015; Smets et al., 2015; Gilmore et al., 2016; Norris et al., 2019). The most common task to measure ANS acuity is dot comparison (De Smedt et al., 2013). In this task, two sets of dots are presented, paired, sequentially, or with an intermixed design (Price et al., 2012). The participants are asked to indicate the larger of two dot arrays without counting. The paired form is the most frequently used dot comparison task because it produces the best psychometric properties (Dietrich et al., 2015). However, even this form has been the focus of much controversy, given that the lack of control of dot arrays’ visual properties (e.g., convex hall, density, etc.) makes it difficult to isolate the effect of non-numerical continuous features from ANS acuity performance during the task (Gebuis and Reynvoet, 2011). Furthermore, performance during dot comparison seems to require additional cognitive processes, particularly inhibitory control, which might also interact with numerical ratio (Clayton and Gilmore, 2015). In the present study, we assessed to what extent non-numerical continuous features and inhibitory control skills predict dot comparison performance in young children.

### 1.1. The role of continuous magnitudes on dot comparison

The rationale behind a dot comparison task is that the difficulty of a trial depends on the ratio between sets of dots; that is, participants become less accurate and slower as differences between the arrays decrease (i.e., it is easier to perceive the difference between 8 vs. 4 than between 8 vs. 7; Piazza et al., 2004; Barth, 2008; Sasanguie et al., 2013; Halberda and Odic, 2015). Hence, measuring ANS acuity using a dot comparison task is constrained by Webber’s law (e.g., Dehaene, 1997; Piazza and Izard, 2009). For the last 10 years, however, researchers have been aware that performance in dot comparison tasks is not only influenced by numerosity ratio but also by the visual properties of the stimuli (Gebuis and Reynvoet, 2011).

Visual features like convex hull or surface area naturally correlate with numerosity (the more numerous a dot array, the larger the convex hull and the surface area), so these features could bias participants’ responses (e.g., Gebuis and Reynvoet, 2012a; Clayton et al., 2015; Smets et al., 2015; DeWind and Brannon, 2016; Norris et al., 2019). To rule out the confound of visual properties, careful control of its effects must be implemented. Generally, two types of trials are designed, congruent vs. incongruent. Visual features positively correlate with numerosity in the congruent trials, while in the incongruent trials, they are manipulated to correlate negatively with numerosity. In general, participants tend to be more accurate and faster on congruent than incongruent trials (e.g., Cappelletti et al., 2014), but this effect is not always significant (e.g., Odic et al., 2013). Gilmore et al. (2016) suggest that differences between congruent and incongruent trials can be explained partly by the lack of control of the stimuli’s visual properties across studies since different algorithms have been used throughout the years.

Two of the most widespread algorithms are the Panamath software (Halberda et al., 2008) and Gebuis and Reynvoet (2011) method. Although both have been used to measure ANS, the results of the tasks created with these two methods show low correlations (Clayton et al., 2015; DeWind and Brannon, 2016), which indicates they are measuring different constructs. Recent studies also demonstrated that Gebuis and Reynvoet’s tasks showed better test–retest reliability than the Panamath task, which presented very low reliability (Clayton et al., 2015). Furthermore, Reynvoet and Gebuis’s algorithm controls for both convex hull and surface area (Norris et al., 2019), which implies that average dot size and density are also controlled for because these two dimensions are highly correlated with surface area (Gebuis and Reynvoet, 2012b). Panamath software only controls for surface area (Clayton et al., 2015; Guillaume et al., 2020), so participants’ responses in Panamath tasks can be influenced by convex hull cues available in incongruent trials and not only by the numerosity of dot arrays. In short, to properly measure ANS, it is crucial to ensure as much as possible that the continuous visual features of the stimuli do not interfere with numerosity during dot comparison. To this end, using the Gebuis and Reynvoet algorithm is a better approximation than the Panama protocol.

### 1.2. Influence of inhibitory control on dot comparison

Even when carefully controlling for all visual properties, individual differences in domain-general cognitive processing can influence dot comparison responses. In particular, congruency effects in dot comparison are thought to rely on inhibitory control (Hurewitz et al., 2006; Nys and Content, 2012; Gilmore et al., 2013; Szucs et al., 2013; Clayton et al., 2015). The rationale is that dot comparison trials work as a Stroop task where participants must attend to relevant information (i.e., numerosity) for responding while ignoring task-irrelevant distractions (i.e., continuous visual features). Thus, participants tend to be less accurate and slower on incongruent trials (ignoring visual features such as convex hull and surface area) than on congruent trials, which may indicate that the first requires additional cognitive demands to process numerical information (Nys and Content, 2012). This implies that decision to dot comparison is not only influenced by numerosity and visual features but also by individual differences in inhibitory control skills. It is relevant to ask, then, to what extent inhibitory control is modulating responses during decisions to incongruent trials.

Up to date, only two studies have directly assessed the contribution of inhibitory control to dot comparison performance in the early stages of numerical skills acquisition (Fuhs et al., 2018; Wilkey et al., 2021). Fuhs et al. (2018) showed that preschool children’s dot comparison performance, specifically in incongruent trial responses, was influenced by a combination of both inhibitory control and cognitive flexibility as indicators of executive function. However, in a second stage, they could not replicate these findings in a subsample of the children. Wilkey et al. (2021) studied the change from the middle to the end of first grade in children’s dot comparison performance and the contribution of executive function to this change. Executive function was measured with the Head, Toes, Knees, and Shoulders (HTKS) task, which requires inhibitory control, among other cognitive processes (e.g., flexibility, self-regulation). Children’s HTKS performance did not predict accuracy change either on congruent or incongruent trials. These two studies’ contradictory findings may result from differences in executive control tasks or statistical approaches. It is also worth noting that both studies used the Panamath protocol, so convex hull variation was not systematically controlled for, which could have affected the responses on incongruent trials. Additionally, both studies failed to isolate inhibitory control from other measures of executive function, so the contribution of inhibitory control alone remains to be tested. Finally, none of the studies added the contribution of numerical ratio as a predictor in the model, which would have allowed testing whether inhibitory control fully or partly explained dot comparison responses in the incongruent trials.

Unlike the above studies, others have only indirectly analysed the relationship between performance in dot comparison and inhibitory control, using these two skills as predictors of math performance (Fuhs and Mcneil, 2013; Gilmore et al., 2013; Keller and Libertus, 2015; Cai et al., 2018). Findings from these studies have not shown a clear pattern; while some have reported significant correlations between dot comparison tasks and inhibitory control (Fuhs and Mcneil, 2013; Gilmore et al., 2013), others have not or found mixed findings (Keller and Libertus, 2015; Cai et al., 2018). Fuhs and McNeil (2013) showed that low-SES preschool children’s dot comparison accuracy correlated with inhibitory control skills, and the correlation was higher between inhibitory control skills and incongruent trials than between inhibitory control skills and congruent trials. Gilmore et al. (2013) found similar results with older children (Age, M = 9.4, SD = 0.6 years), where dot comparison accuracy was found to share its predicted variance on mathematics with inhibitory control, indicating that both variables were related. Keller and Libertus (2015) conducted two experiments with young children (Age, M = 55 months, SD = 5 months). The first one did not find significant correlations between dot comparison accuracy and inhibitory control skills, but the second one did. The authors suggested that differences in inhibitory control tasks used in the two experiments could explain the different patterns. It is important to note that in this study, the strongest correlation was between inhibition and congruent trials, which differs from Fuhs and Mcneil (2013)‘s findings. Finally, Cai et al. (2018) found a significant correlation between dot comparison accuracy (as the overall score included congruent and incongruent trials) and inhibitory control in kindergarteners, but they did not in second and third-grade children.

In synthesis, there is no consensus across studies; while some have found significant correlations between dot comparison performance and inhibitory control, others have not. Those that found overall correlations also diverged when data analysis was conducted separately by type of trial (congruent vs. incongruent). Some displayed a stronger correlation between inhibitory control and incongruent trials, while others found a stronger correlation between inhibitory control and congruent trials. It is important to note that most of these studies used Panamath software except Gilmore et al. (2013) which used Gebuis and Reynvoet algorithm. The latter, however, did not explore the effect of inhibitory control separately by type of trial. In sum, the inconsistency found across studies requires additional evidence to clearly understand the role of inhibitory control on ANS measures, particularly in the dot comparison task.

### 1.3. The current study

In the present study, we analysed the influence of inhibitory control, non-numerical continuous features, and numerical ratio on dot comparison performance of preschool children by using linear mixed effect models. To control for the effects of visual properties, we used the Gebuis and Reynvoet algorithm (Gebuis and Reynvoet, 2011) because it can successfully control for both convex hull and surface area, unlike the Panama software (Halberda et al., 2008), which can only control for surface area. Unlike Fuhs et al. (2018) and Wilkey et al. (2021), we included fully congruent and incongruent trials, as well as “surface area incongruent” trials, which have also demonstrated to trigger congruency effects in young children (Gilmore et al., 2016). Finally, considering the low statistical power identified by Chen and Li (2014) in most studies in this field, we used a larger sample of children than in all previous studies.

Based on the above information, we hypothesise a numerical ratio effect, due to the approximate nature of the dot comparison task, with children being less accurate as the ratio increases, in line with the large body of previous empirical evidence (e.g., Dehaene, 1992; Cantlon and Brannon, 2006; Libertus et al., 2007; Soltész et al., 2010; Agrillo et al., 2013; Sasanguie et al., 2013). Secondly, given that differences in performance across trial types depend on the visual properties of the stimuli (Gebuis and Reynvoet, 2012a,b), we expect children to be more accurate on trials that are congruent with the visual properties of the stimuli than on incongruent ones (e.g., Clayton et al., 2015) and particularly inaccurate when all visual cues are negatively correlated with numerosity (e.g., Clayton et al., 2019). Thirdly, provided that recent studies with adults suggest that the numerosity ratio effect is also influenced by the continuous visual properties of the stimuli (Braham et al., 2018; Reynvoet et al., 2021), we expect the numerosity ratio effect to be larger in incongruent trials than in congruent ones. As stated earlier, the congruency effect has been interpreted as the effort to suppress the response based on visual features to respond according to the numerosity of the arrays (Szucs et al., 2013), so we also predict that inhibitory control skills will influence performance in incongruent trials but not in congruent ones. As very few studies have included inhibitory control as a predictor of congruent and incongruent dot comparison accuracy, we cannot formulate a clear hypothesis about the influence of ratio and inhibitory control across trial types.

## 2. Materials and methods

### 2.1. Participants

Participants included 517 preschool children (Age, M = 61.14 months, SD = 3.40 months), 268 girls (51.8%) who attended 13 urban schools located in Concepción (Chile). This is the first sample of a longitudinal study currently underway. Children came from heterogeneous socioeconomic backgrounds. The Chilean educational legislation assigns each school a school vulnerability index (SVI) published annually by the National Board of School Aid and Scholarships (JUNAEB). This index represents the percentage of students in each school categorised as a priority based on their poverty level and school failure risk. Using SVI data for 2019 and based on criteria defined by the Chilean Ministry of Education (Ministerio de Educación de Chile, 2019), schools and students were distributed as follows: (a) High-SVI (more than 70% categorised as a priority), seven schools, 265 children (51%); Medium-SVI (30–70% categorised as a priority), four schools, 182 children (35%); and (c) low-SVI (below 35% categorised as a priority), two schools, 70 children (13%).

### 2.2. Materials

All measures were administered using a 15′ touchscreen laptop. Participants and examiners were required to put on a set of headphones to listen to the instructions together. Children sat approximately 30 cm away from the laptop screen to begin the task.

#### 2.2.1. Inhibitory control

Sun-moon Stroop task (Archibald and Kerns, 1999). This is a variant of the original day/night Stroop task (Gerstadt et al., 1994). Two sets of 24 pictures of suns and moons arranged randomly were displayed in 4 rows and six columns. In the first set/condition (congruent) children were asked to say “/sun/” [/sol/] if they saw a picture of a sun, and “/moon/” [/luna/] if they saw a picture of a moon. In the second set/condition (incongruent), they were asked to say “/sun/” for a picture of a moon a “/moon/” for a picture of a sun. The children did some practice trials in each block (congruent and incongruent) to ensure that they understood the task. Children were asked to respond as quickly as possible in both conditions and had a 45-s time limit. The score used was the total number of correct test trial responses in the second set.

Grass–snow task (Carlson and Moses, 2001). In this task, children were required to touch one of two solid color patches (green and white) presented in the centre of the computer screen. Two sets of 20 pairs of green/white patches were presented simultaneously side-by-side. In the first set, children were asked to touch the white color patch when they heard “snow” (nieve) and to touch the green color patch when they heard “grass” (pasto). In the second set, children were required to touch the white color patch when they heard “grass” and to touch the green color patch when they heard “snow.” The score used was the total number of correct responses in the incongruent condition.

Children’s scores in the two inhibitory control tasks were significantly correlated (*r* = 0.29**), so they were converted into z-scores and summed to create a composite inhibitory control measure for all analyses.

#### 2.2.2. Dot comparison task

The present task consisted of 6 practice and 48 experimental trials. The ratio between dot arrays was 0.5, 0.6, 0.7 and 0.8. The number represented ranged from 5 to 28. The stimuli were generated following the algorithm of Gebuis and Reynvoet. Three sets of dots were created: (a) fully congruent, that is convex hull and surface area, together with other visual cues (total circumference, density, and dot size) positively correlated with numerosity; (b) fully incongruent, all visuals negatively correlated with numerosity, and; (c) surface area incongruent, the convex hull was positively correlated with numerosity and surface area negatively correlated with numerosity. To design de present study, Children were asked to sit comfortably in front of a touchscreen laptop computer. For each trial, children saw a fixation point on the screen for 1,000 ms followed by the dot arrays presented for 1,500 ms, and a blank screen until response. They were required to select the more numerous arrays by touching the arrays on the laptop screen as quickly as possible. Accuracy and RT were recorded. The score used in the present study was accuracy because it can reliably index the precision of the ANS (Inglis and Gilmore, 2014).

### 2.3. Procedure

The study received prior approval from the Ethics Committee of the Universidad Católica del Maule. Before the assessment, parent careers of all participants provided written informed consent for their child to take part. Children were asked if they wanted to participate before the first session in the presence of a teacher, if they agreed, the examiner would write down the child’s name in the child’s written consent. If the child were able to write their name by himself, they were allowed to. The present tasks were administered in a fixed order and were part of a larger battery of cognitive and numerical skills that extended over three sessions. The study took place in a quiet room within the school, but to ensure that the children heard the instructions loud and clear we required them to use headphones. At the same time, to ensure proper monitoring of each child throughout the tasks, the examiner was also required to wear headphones. Each session lasted approximately 20 min, and children had to complete four or five tasks. The tasks involved assessment of numerical processing, counting, working memory and inhibitory control and one standardized measure. The order of the tasks and their distribution across sessions was designed so that sessions were balanced in terms of time and effort; and within each session, so that more demanding tasks were interspersed with less demanding tasks.

## 3. Results

### 3.1. Data analysis

Different 2-level logistic mixed effects models were estimated to predict individual participants’ accuracy on each trial of the dot comparison task. At level 1 (or trial level), numerical ratio was entered as predictor and the three trial types serving as the comparison group. At level 2 (or participant-level), inhibitory control tasks were entered as predictors. Mean and standard deviation of the predictors at level 1 and level 2 are presented in Table 1.

**Table 1:** Mean and standard deviation of accuracy (percentage) in the dot comparison task.

*Level 1 predictors*			
Trial Type	Ratio	M	SD
Fully congruent	Easy	0.86	0.35
	Medium	0.86	0.35
	Hard	0.81	0.39
	Very hard	0.82	0.38
Surface area incongruent	Easy	0.68	0.47
	Medium	0.66	0.47
	Hard	0.63	0.48
	Very hard	0.54	0.5
Fully incongruent	Easy	0.63	0.48
	Medium	0.51	0.5
	Hard	0.44	0.5
	Very hard	0.39	0.49
*Level 2 predictors*			
Inhibitory control tasks		M	SD
Sun-Moon Stroop incongruent (accuracy)		16.56	6.66
Grass-Snow incongruent (accuracy)		12.7	6.15
Inhibition (composite score)		0.02	1.59

We conducted the analyses in two stages. First, we calculated one model with all level-1 and level-2 predictors entered without any interaction. Then, we entered the interaction between all predictors (trial type, inhibitory control tasks and numerical ratio) to test whether children’s dot comparison task performance varied across trial type as a function of their inhibitory control skills. We also tested whether the expected negative relationship between dot comparison accuracy and ratio (as the ratio increased the comparisons become more difficult) varied according to trial type and children’s inhibitory control skills. Finally, we compared the two models in order to identify the model that best fit our data using the anova function in software R versión 3.3.5 (R Core Team, 2017) and ULLRToolbox (Hernández and Betancort, 2018). The numerical ratio was introduced as a continuous variable in the analyses, but for ease of visualization and interpretation, we split it into four bins, with the following numerical ratio: “easy” = 0.5; “medium” > 0.5 and = <0.65; “hard” > 0.65 and < 0.75; “very hard” = > 0.75.

### 3.2. Prediction of dot comparison accuracy

Performance on the dot comparison tasks was quantified as the percentage of correct responses. Children were more accurate in the fully congruent trials (around 85%) than in the incongruent ones (fully and surface area incongruent), irrespective of the numerical ratio. In general terms, children were more accurate on smaller ratios (easy or medium) than on larger ratios (hard or very hard). In the fully incongruent trials, children responded above chance only in the comparison with the smallest ratio or easiest comparison. Accuracy to surface-area incongruent trials was above chance except for trials that used the largest ratio (very hard trials; see Table 1).

The results of the two 2-level logistic mixed effects model comparisons showed significant differences (see Table 2). The likelihood-ratio test indicated that the model with interactions provided a better fit to the data than the model without interactions. We also examined Akaike Information Criterion (AIC) and the Bayesian Information Criterion (BIC), which are complementary fit indexes. Overall, lower AIC and BIC values indicate better model fit. Model 2 had lower AIC but higher BIC than model 1. However, given that BIC penalizes adding complexity to models more than AIC (Sober, 2002) and that the likelihood-ratio test showed model 2 improved prediction, model 2 was selected for report over model 1.

**Table 2:** Models’ comparison with anova function.

	df	AIC	BIC	log-Likelihood	χ^2^	df_χ_^2^	*p*
Model 1	8	29,171	29,236	−14,577			
Model 2	15	29,121	29,243	−14,546	63.55	7	<0.001

**Figure 1:**
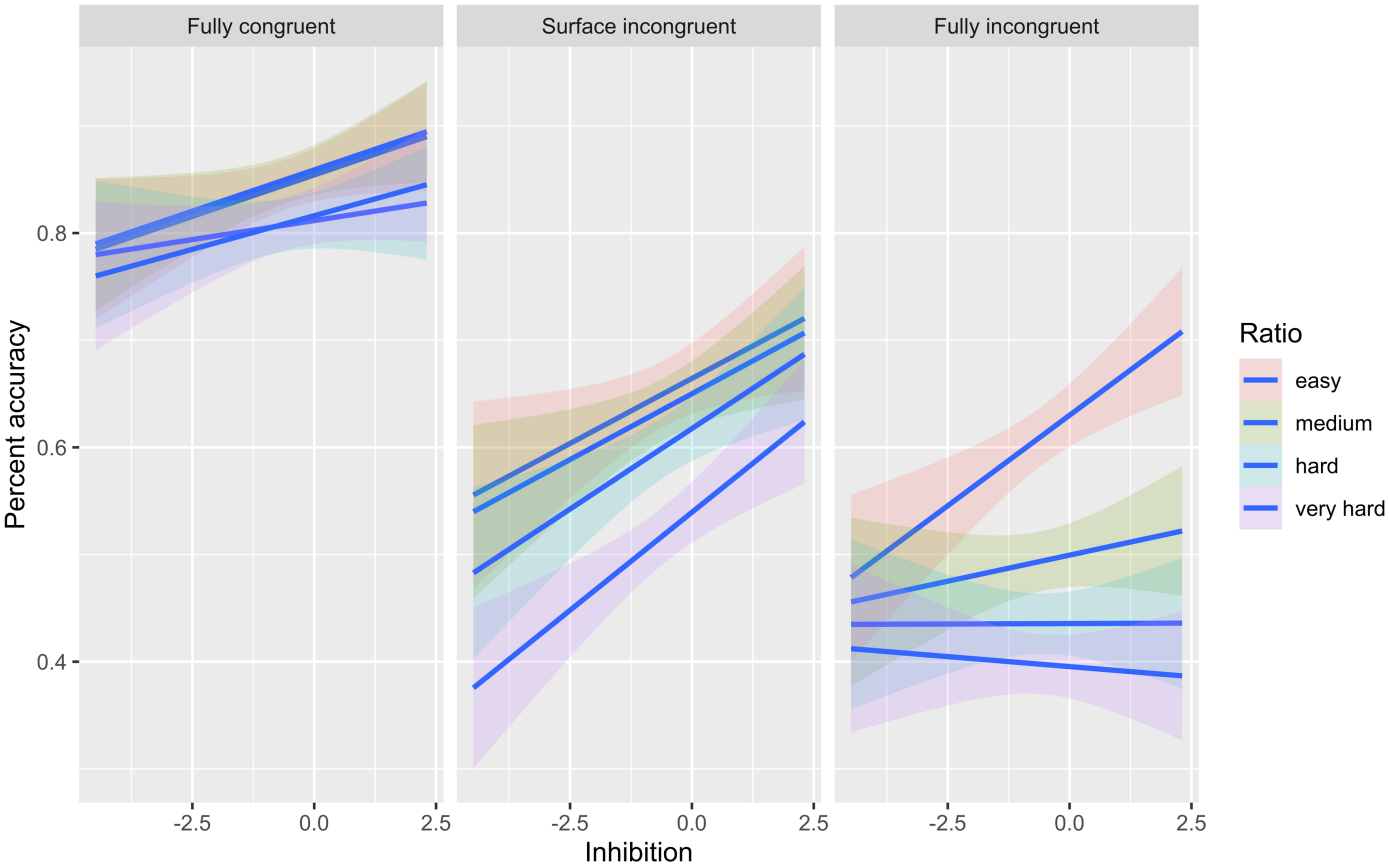
Interaction effect between Inhibitory control, type of trial, and ratio on accuracy.

In model 1, all level-1 and level-2 predictors made significant contributions to accuracy; however, in model 2, all the main effects were involved in double and triple interactions (see Table 3). The first double interaction between ratio and the type of trial (Ratio: Fully incongruent) revealed that the relationship between ratio and dot comparison accuracy depends on trial type. The coefficient was negative, indicating a diminishing effect, which implies that as the ratio increased (became harder to solve), dot comparison accuracy decreased, with this relationship being significantly stronger for fully incongruent trials than for other trial types. The second double interaction between inhibitory control and type of trial (Fully incongruent: Inhibition) showed that the relationship between inhibition and dot comparison accuracy depends on trial type. The coefficient was positive, indicating an enhancing effect, which means that as accuracy in the inhibition tasks increased, so did dot comparison accuracy, and the relationship became stronger for fully incongruent trials. Next, we examined the triple interaction between ratio, type of trial, and inhibitory control (Ratio: Fully incongruent: Inhibition). The relationship between dot comparison accuracy and inhibitory control decreased as the ratio increased for fully incongruent trials. There was no change as a function of ratio in the relationship between accuracy and inhibitory control tasks in fully congruent and surface area incongruent trials (see Figure 1).

**Table 3:** Results of the two-level logistic mixed models predicting dot’s comparison accuracy.

	Estimate	SE	*t*	*p*
***Model 1***				
Intercept	1.138	0.062	18.24	<0.001
Ratio	−0.461	0.092	−5.01	<0.001
Fully incongruent	−0.346	0.024	−14.16	<0.001
Surface incongruent	−0.210	0.024	−8.60	<0.001
Inhibition	0.019	0.003	5.99	<0.001
***Model 2***				
Intercept	0.935	0.096	9.74	0.001
Ratio	−0.149	0.146	−1.02	0.312
Fully incongruent	0.084	0.136	0.62	0.537
Surface incongruent	−0.034	0.136	−0.25	0.800
Inhibition	0.014	0.018	0.75	0.452
Ratio: Fully incongruent	−0.663	0.206	−3.22	<0.01
Ratio: Surface incongruent	−0.271	0.206	−1.32	0.195
Ratio: Inhibition	−0.001	0.028	−0.049	0.961
Fully incongruent: Inhibition	0.085	0.026	3.24	<0.01
Surf incongruent: Inhibition	−0.001	0.026	−0.050	0.961
Ratio: Fully incongruent: Inhibition	−0.131	0.040	−3.31	<0.01
Ratio: Surf incongruent: Inhibition	0.032	0.039	0.83	0.408

Surf_incong = surface-incongruent trials; Fully_incong = Fully-incongruent trials.

## 4. Discussion

In the current study, we analysed the contribution of inhibitory control, non-numerical continuous features, and numerical ratio on the dot comparison performance of preschool children. ANS has been the focus of numerous studies due to its possible role in the development of numerical and mathematical skills and even as a deficit in the case of atypical development (specific learning disabilities in math). The rationale in the present study was that dot comparison was the most used experimental task to measure ANS, although it could be influenced by aspects that go beyond estimation precision. Therefore, it was important to find out whether the relationship between dot comparison and mathematical performance was not confounded with inhibitory control and non-numerical features processing.

The results of our study partially confirmed the hypothesis about the numerical ratio effect given the approximate nature of the dot comparison task. Performance on dot comparison tasks is characterized by a ratio effect (e.g., Halberda and Feigenson, 2008; Price et al., 2012; Agrillo et al., 2013; Sasanguie et al., 2013), that is, as the differences between two numerosities becomes smaller (more difficult comparisons), participants are less accurate than when the differences are larger (easier comparisons). In the present study, we found that the ratio effect was moderated by trial type, as expected. There was a ratio effect on accuracy for fully incongruent trials, but not for congruent ones. A similar interaction between ratio and congruency was found in recent studies with adults (e.g., Braham et al., 2018; Reynvoet et al., 2021). As in Reynvoet et al.’s study, the present finding may be explained by a ceiling effect in fully congruent trials and the low accuracy in incongruent trials, especially in fully incongruent ones. High accuracy in fully congruent trials has been reported previously in other studies (e.g., Gilmore et al., 2013; Wilkey et al., 2021). Braham et al. (2018) suggested that when trials are difficult due to the ratio being high (small differences between the two quantities), children may use other cues to solve the comparison. In the fully incongruent trials, this strategy decreased accuracy. The lack of a significant ratio effect on congruent trials suggests that when continuous properties of stimuli and numerosities correlate, selecting the correct dot array is triggered by continuous properties rather than by numerosities.

Our second hypothesis stating that children would perform better in congruent than incongruent trials was confirmed. Children answered fully congruent trials significantly more accurately than incongruent trials. They were particularly inaccurate in fully incongruent trials, with performance falling below chance level across all trials except for those that were categorised as the easiest according to their ratio. These findings suggest that children do not process numerical information independently of the continuous features of dot arrays in line with previous studies (e.g., Gebuis and Reynvoet, 2012a,b; Clayton et al., 2019). Although some studies have failed to replicate this effect in children (e.g., Odic et al., 2013; Odic, 2018), they did not control for convex hull, so children could use this cue to solve the task. In the present study, we created the stimuli using Gebuis and Reynvoet (2011) algorithm, which controls for both convex hull and surface area, including surface-incongruent trials in which convex hull correlates positively with numerosity. Accuracy in surface-incongruent trials was lower than in fully congruent trials but higher than in fully incongruent ones. This finding suggests that children used convex hull information to decide which dot array had the most dots, unlike in incongruent trials, where they were unable to use any continuous features during item selection. Wilkey et al. (2021) suggested that the predictive weight of continuous magnitudes decreases with age, while the weight of numerical information increases, so it is expected that preschool children rely more on continuous magnitudes than on numerosity.

In our third hypothesis, we expected a significant influence of inhibitory control skills on accuracy for incongruent trials given that the congruency effect in dot comparison tasks is interpreted as the cost of inhibiting information of continuous properties to process discrete quantities (Nys and Content, 2012; Fuhs and Mcneil, 2013; Gilmore et al., 2013; Szucs et al., 2013). The current findings supported our hypothesis. This means that as the inhibitory control skills increased, accuracy in incongruent trials also increased. The three-way interaction found between inhibitory control, accuracy, and ratio suggests the relationship between inhibitory control and dot comparison accuracy is moderated by ratio. The relationship between inhibitory control and accuracy decreases as the ratio increases (comparison becomes more challenging). When trials were “easy” or “medium,” the relationship between inhibitory control and accuracy was positive, however, when trials were “hard” or “very hard” the relationship declined. The explanation for this result may be linked to the fact that children tend to use continuous cues more than numerosity when the ratios are large. Numerosity in this trial type is much less prominent than continuous properties, which yields more robust representations. Children could mainly use continuous properties (dominant response) due to the difficulty of extracting quantitative information, which implies that they would not need to use inhibitory control skills. However, this interpretation has to be taken with caution, since children’s performance in fully incongruent trials was below chance in the hardest trials.

The present findings converge with those of Fuhs et al. (2018) and diverge from those of Wilkey et al. (2021). Fuhs et al. found a significant relationship between dots comparison accuracy for incongruent trials and a composite measure of inhibitory control and flexibility. While Wilkey et al. found no significant influence of inhibitory control on the accuracy of congruent or incongruent dots comparison. Our results are more comparable to those of Fuhs et al.’s than to those of Wilkey et al.’s because the former conducted the study with children of a similar age to our participants and with similar inhibitory control tasks; these included the Day-Night task (Gerstadt et al., 1994) and the Dimensional Change Card Sort (DCCS; Zelazo, 2006). By contrast, in the study by Wilkey et al., children were older (first graders) than our participants and had already received formal mathematical instruction. It is shown that the interference from the continuous visual properties is stronger in younger than in older children (Clayton et al., 2019), and this could be related to the development of inhibitory control skills across ages (Morton, 2010; Petersen et al., 2016). Thus, the lack of relationship between inhibitory control and dot comparison accuracy could be explained partially because of children’s developmental stage. On top of that, they used HTKS as a measure of inhibitory control, which involves many other cognitive processes in addition to inhibitory control.

There are some limitations in the current study. Children displayed a very low success rate in the fully incongruent condition, which we attributed to their age. Future work should look into this condition in older children in order to better understand the role of continuous magnitudes and inhibitory control in fully incongruent trials. Finally, this study was cross-sectional, so it does not inform of children’s trajectories. Subsequent studies should incorporate longitudinal measures to better understand the relationship among the above factors.

## 5. Conclusion

The results of this study show that preschool children rely more on continuous magnitudes than numerosity in dot comparison tasks. They cannot make a reliable estimation when continuous properties of dots arrays are all incongruent with numerosity. When not all continuous magnitudes are incongruent with numerosities, like in surface area incongruent trials, children use the congruent continuous properties simultaneously with numerosity to respond correctly. The greater children’s inhibitory control, the more able they are to respond based on numerosity in fully incongruent trials, except in those with higher ratios (hard to solve). In these trial types, children are not able to extract numerical information, so they simply use the most salient information, which happens to be continuous properties. The above support the competing processes account given that our findings suggest that both ANS and inhibitory control skills influence performance in dot comparison tasks.

## Data availability statement

The raw data supporting the conclusions of this article will be made available by the authors, without undue reservation.

## Ethics statement

The studies involving human participants were reviewed and approved by Ethics Committee of the Universidad Católica del Maule. Written informed consent to participate in this study was provided by the participants’ legal guardian/next of kin.

## Author contributions

CR: conceptualization and methodology. RF: methodology and software. All authors contributed to writing, editing, reviewing, and approving the final manuscript. All authors contributed to the article and approved the submitted version.

## Funding

This work was funded by ANID through Fondecyt Regular grant No. 1191589 and Millennium Science Initiative Program - NCS2022-026.

## Acknowledgments

We thank, schools, teachers and especially all children for their participation.

## Conflict of interest

The authors declare that the research was conducted in the absence of any commercial or financial relationships that could be construed as a potential conflict of interest.

## Publisher’s note

All claims expressed in this article are solely those of the authors and do not necessarily represent those of their affiliated organizations, or those of the publisher, the editors and the reviewers. Any product that may be evaluated in this article, or claim that may be made by its manufacturer, is not guaranteed or endorsed by the publisher.

## References

[ref1] Agrillo C. Piffer L. Adriano A. (2013). Individual differences in non-symbolic numerical abilities predict mathematical achievements but contradict ATOM. Behav. Brain Funct. 9, 26–14. doi: 10.1186/1744-9081-9-26, PMID: 23815866 PMC3711901

[ref2] Archibald S. J. Kerns K. A. (1999). Identification and description of new tests of executive functioning in children. Child Neuropsychol. 5, 115–129. doi: 10.1076/CHIN.5.2.115.3167

[ref3] Barth H. C. (2008). Judgments of discrete and continuous quantity: an illusory Stroop effect. Cognition 109, 251–266. doi: 10.1016/J.COGNITION.2008.09.002, PMID: 18973877

[ref4] Braham E. J. Elliott L. Libertus M. E. (2018). Using hierarchical linear models to examine approximate number system acuity: the role of trial-level and participant-level characteristics. Front. Psychol. 9:2081. doi: 10.3389/fpsyg.2018.02081, PMID: 30483169 PMC6240605

[ref5] Cai D. Zhang L. Li Y. Wei W. Georgiou G. K. (2018). The role of approximate number system in different mathematics skills across grades. Front. Psychol. 9:1733. doi: 10.3389/fpsyg.2018.01733, PMID: 30279672 PMC6153330

[ref6] Cantlon J. F. Brannon E. M. (2006). Shared system for ordering small and large numbers in monkeys and humans. Psychol. Sci. 17, 401–406. doi: 10.1111/j.1467-9280.2006.01719.x, PMID: 16683927

[ref7] Cappelletti M. Didino D. Stoianov I. Zorzi M. (2014). Number skills are maintained in healthy ageing. Cogn. Psychol. 69, 25–45. doi: 10.1016/j.cogpsych.2013.11.004, PMID: 24423632

[ref01] Carlson S. M. Moses L. J. (2001). Individual differences in inhibitory control and children’s theory of mind. Child Development 72, 1032–1053. doi: 10.1111/1467-8624.00333 11480933

[ref8] Chen Q. Li J. (2014). Association between individual differences in non-symbolic number acuity and math performance: a meta-analysis. Acta Psychol. 148, 163–172. doi: 10.1016/J.ACTPSY.2014.01.016, PMID: 24583622

[ref9] Clayton S. Gilmore C. (2015). Inhibition in dot comparison tasks. ZDM 47, 759–770. doi: 10.1007/s11858-014-0655-2

[ref10] Clayton S. Gilmore C. Inglis M. (2015). Dot comparison stimuli are not all alike: the effect of different visual controls on ANS measurement. Acta Psychol. 161, 177–184. doi: 10.1016/j.actpsy.2015.09.007 26408864

[ref11] Clayton S. Inglis M. Gilmore C. (2019). Developmental differences in approaches to nonsymbolic comparison tasks. Q. J. Exp. Psychol. 72, 436–445. doi: 10.1177/1747021818755296, PMID: 29419356

[ref12] De Smedt B. Noël M. P. Gilmore C. Ansari D. (2013). How do symbolic and non-symbolic numerical magnitude processing skills relate to individual differences in children’s mathematical skills? A review of evidence from brain and behavior. Trends in Neuroscience and Education 2, 48–55. doi: 10.1016/j.tine.2013.06.001

[ref13] Dehaene S. (1992). Varieties of numerical abilities. Cognition 44, 1–42. doi: 10.1016/0010-0277(92)90049-N 1511583

[ref14] Dehaene S. (1997). The number sense: How the mind creates mathematics. Oxford: Oxford University Press.

[ref15] Desoete A. Ceulemans A. Weerdt F. De Pieters S. (2012). Can we predict mathematical learning disabilities from symbolic and non-symbolic comparison tasks in kindergarten? Findings from a longitudinal study. 82, 64–81. doi: 10.1348/2044-8279.002002 21199482

[ref16] DeWind N. K. Brannon E. M. (2016). Significant inter-test reliability across approximate number system assessments. Front. Psychol. 7:310. doi: 10.3389/fpsyg.2016.00310, PMID: 27014126 PMC4781867

[ref17] Dietrich J. F. Huber S. Nuerk H. C. (2015). Methodological aspects to be considered when measuring the approximate number system (ANS) - a research review. Front. Psychol. 6, 1–14. doi: 10.3389/fpsyg.2015.00295, PMID: 25852612 PMC4362052

[ref18] Duncan G. J. Dowsett C. J. Claessens A. Magnuson K. Huston A. C. Klebanov P.. (2007). School readiness and later achievement. Dev. Psychol. 43, 1428–1446. doi: 10.1037/0012-1649.43.6.1428 18020822

[ref20] Fazio L. K. Bailey D. H. Thompson C. A. Siegler R. S. (2014). Relations of different types of numerical magnitude representations to each other and to mathematics achievement. J. Exp. Child Psychol. 123, 53–72. doi: 10.1016/J.JECP.2014.01.013, PMID: 24699178

[ref21] Feigenson L. Dehaene S. Spelke E. (2004). Core systems of number. Trends Cogn. Sci. 8, 307–314. doi: 10.1016/j.tics.2004.05.002 15242690

[ref22] Fuhs M. W. Mcneil N. M. (2013). ANS acuity and mathematics ability in preschoolers from low-income homes: contributions of inhibitory control. Dev. Sci. 16, 136–148. doi: 10.1111/desc.12013, PMID: 23278935

[ref23] Fuhs M. W. Nesbitt K. T. O’Rear C. D. (2018). Approximate number system task performance: associations with domain-general and domain-specific cognitive skills in young children. J. Numerical Cognition 4, 590–612. doi: 10.5964/jnc.v4i3.141

[ref24] Gebuis T. Reynvoet B. (2011). Generating nonsymbolic number stimuli. Behav. Res. Methods 43, 981–986. doi: 10.3758/s13428-011-0097-5 21512872

[ref25] Gebuis T. Reynvoet B. (2012a). The role of visual information in numerosity estimation. PLoS One 7:e37426. doi: 10.1371/journal.pone.0037426, PMID: 22616007 PMC3355123

[ref26] Gebuis T. Reynvoet B. (2012b). The interplay between nonsymbolic number and its continuous visual properties. J. Exp. Psychol. Gen. 141, 642–648. doi: 10.1037/A0026218, PMID: 22082115

[ref27] Gerstadt C. L. Hong Y. J. Diamond A. (1994). The relationship between cognition and action: performance of children 3 1/2-7 years old on a Stroop-like day-night test. Cognition 53, 129–153. doi: 10.1016/0010-0277(94)90068-X, PMID: 7805351

[ref28] Gilmore C. Attridge N. Clayton S. Cragg L. Johnson S. Marlow N.. (2013). Individual differences in inhibitory control, not non-verbal number acuity, correlate with mathematics achievement. PLoS One 8:7374. doi: 10.1371/journal.pone.0067374, PMID: 23785521 PMC3681957

[ref29] Gilmore C. Cragg L. Hogan G. Inglis M. (2016). Congruency effects in dot comparison tasks: convex hull is more important than dot area. J. Cogn. Psychol. 28, 923–931. doi: 10.1080/20445911.2016.1221828, PMID: 28163886 PMC5213839

[ref30] Guillaume M. Schiltz C. Van Rinsveld A. (2020). NASCO: a new method and program to generate dot arrays for non-symbolic number comparison tasks. J. Numerical Cognition 6, 129–147. doi: 10.5964/JNC.V6I1.231

[ref31] Halberda J. Feigenson L. (2008). Developmental change in the acuity of the “number sense”: the approximate number system in 3-, 4-, 5-, and 6-year-olds and adults. Dev. Psychol. 44, 1457–1465. doi: 10.1037/A0012682, PMID: 18793076

[ref32] Halberda J. Mazzocco M. M. M. Feigenson L. (2008). Individual differences in non-verbal number acuity correlate with maths achievement. Nature 455, 665–668. doi: 10.1038/nature07246, PMID: 18776888

[ref33] Halberda J. Odic D. (2015). The precision and internal confidence of our approximate number thoughts. Mathematical cognition and learning 1, 305–333. doi: 10.1016/B978-0-12-420133-0.00012-0

[ref34] Hernández J. A. Betancort M. (2018). ULLRtoolbox. Retrieved 10 junio 2019, from https://sites.google.com/site/ullrtoolbox/

[ref35] Holloway I. D. Ansari D. (2009). Mapping numerical magnitudes onto symbols: the numerical distance effect and individual differences in children’s mathematics achievement. J. Exp. Child Psychol. 103, 17–29. doi: 10.1016/j.jecp.2008.04.001, PMID: 18513738

[ref36] Hurewitz F. Gelman R. Schnitzer B. (2006). Sometimes area counts more than number. Proceedings of the National Academy of Sciences 103, 19599–19604. doi: 10.1073/pnas.0609485103, PMID: 17159143 PMC1748271

[ref37] Inglis M. Gilmore C. (2014). Indexing the approximate number system. Acta Psychol. 145, 147–155. doi: 10.1016/j.actpsy.2013.11.009 24361686

[ref38] Iuculano T. Tang J. Hall C. W. B. Butterworth B. (2008). Core information processing deficits in developmental dyscalculia and low numeracy. Dev. Sci. 11, 669–680. doi: 10.1111/J.1467-7687.2008.00716.X, PMID: 18801122

[ref39] Izard V. Sann C. Spelke E. S. Streri A. (2009). Newborn infants perceive abstract numbers. Proc. Natl. Acad. Sci. 106, 10382–10385. doi: 10.1073/pnas.0812142106, PMID: 19520833 PMC2700913

[ref40] Keller L. Libertus M. (2015). Inhibitory control may not explain the link between approximation and math abilities in kindergarteners from middle class families. Front. Psychol. 6, 1–11. doi: 10.3389/fpsyg.2015.00685, PMID: 26052306 PMC4440905

[ref41] Kolkman M. E. Kroesbergen E. H. Leseman P. P. M. (2013). Early numerical development and the role of non-symbolic and symbolic skills. Learn. Instr. 25, 95–103. doi: 10.1016/j.learninstruc.2012.12.001

[ref42] Korpershoek H. Kuyper H. van Der Werf G. Bosker R. (2011). Who succeeds in advanced mathematics and science courses? Br. Educ. Res. J. 37, 357–380. doi: 10.1080/01411921003671755

[ref43] Libertus M. E. Brannon E. M. (2009). Behavioral and neural basis of number sense in infancy. Curr. Dir. Psychol. Sci. 18, 346–351. doi: 10.1111/j.1467-8721.2009.01665.x, PMID: 20419075 PMC2857350

[ref44] Libertus M. E. Feigenson L. Halberda J. (2013). Is approximate number precision a stable predictor of math ability? Learn. Individ. Differ. 25, 126–133. doi: 10.1016/j.lindif.2013.02.001, PMID: 23814453 PMC3692560

[ref45] Libertus M. E. Woldorff M. G. Brannon E. M. (2007). Electrophysiological evidence for notation independence in numerical processing. Behavioral and Brain Function. 3, 1–15. doi: 10.1186/1744-9081-3-1, PMID: 17214890 PMC1781950

[ref46] Mazzocco M. M. Feigenson L. Halberda J. (2011b). Preschoolers’ precision of the approximate number system predicts later school mathematics performance. PLoS One 6:23749. doi: 10.1371/journal.pone.0023749, PMID: 21935362 PMC3173357

[ref47] Mazzocco M. M. M. Feigenson L. Halberda J. (2011a). Impaired acuity of the approximate number system underlies mathematical learning disability (dyscalculia). Child Dev. 82, 1224–1237. doi: 10.1111/j.1467-8624.2011.01608.x, PMID: 21679173 PMC4411632

[ref48] Ministerio de Educación de Chile. (2019). Junta Nacional de Auxilio Escolar y Becas (JUNAEB). Available online at: https://www.junaeb.cl/como-funciona-el-sinae (accessed March 19, 2020).

[ref49] Morton J. (2010). Understanding genetic, neurophysiological, and experiential influences on the development of executive functioning: the need for developmental models. WIREs Cognitive Science 1, 709–723. doi: 10.1002/wcs.87, PMID: 26271655

[ref50] Mussolin C. De Volder A. Grandin C. Schlögel X. Nassogne M. C. Noël M. P. (2010). Neural correlates of symbolic number comparison in developmental dyscalculia. J. Cogn. Neurosci. 22, 860–874. doi: 10.1162/JOCN.2009.21237, PMID: 19366284

[ref51] Norris J. E. Clayton S. Gilmore C. Inglis M. Castronovo J. (2019). The measurement of approximate number system acuity across the lifespan is compromised by congruency effects. Q. J. Exp. Psychol. 72, 1037–1046. doi: 10.1177/1747021818779020, PMID: 29747553

[ref52] Nys J. Content A. (2012). Judgement of discrete and continuous quantity in adults: number counts! Q. J. Exp. Psychol. 65, 675–690. doi: 10.1080/17470218.2011.619661, PMID: 22054280

[ref53] Odic D. (2018). Children’s intuitive sense of number develops independently of their perception of area, density, length, and time. Dev. Sci. 21, 1–15. doi: 10.1111/desc.12533, PMID: 28497512

[ref54] Odic D. Libertus M. E. Feigenson L. Halberda J. (2013). Developmental change in the acuity of approximate number and area representations. Dev. Psychol. 49, 1103–1112. doi: 10.1037/a0029472, PMID: 22889394 PMC4388157

[ref55] Parsons S. Bynner J. (1997). Numeracy and employment. Education + Training 39, 43–51. doi: 10.1108/00400919710164125

[ref56] Petersen I. T. Hoyniak C. P. McQuillan M. E. Bates J. E. Staples A. D. (2016). Measuring the development of inhibitory control: the challenge of heterotypic continuity. Dev. Rev. 40, 25–71. doi: 10.1016/j.dr.2016.02.001, PMID: 27346906 PMC4917209

[ref57] Piazza M. Izard V. (2009). How humans count: Numerosity and the parietal cortex. Neuroscientist 15, 261–273. doi: 10.1177/1073858409333073, PMID: 19436075

[ref58] Piazza M. Izard V. Pinel P. Le Bihan D. Dehaene S. (2004). Tuning curves for approximate Numerosity in the human intraparietal sulcus. Neuron 44, 547–555. doi: 10.1016/J.NEURON.2004.10.014, PMID: 15504333

[ref59] Praet M. Titeca D. Ceulemans A. Desoete A. (2013). Language in the prediction of arithmetics in kindergarten and grade 1. Learn. Individ. Differ. 27, 90–96. doi: 10.1016/j.lindif.2013.07.003

[ref61] Price G. R. Holloway I. Räsänen P. Vesterinen M. Ansari D. (2007). Impaired parietal magnitude processing in developmental dyscalculia. Curr. Biol. 17, R1042–R1043. doi: 10.1016/J.CUB.2007.10.013, PMID: 18088583

[ref62] Price G. R. Palmer D. Battista C. Ansari D. (2012). Nonsymbolic numerical magnitude comparison: reliability and validity of different task variants and outcome measures, and their relationship to arithmetic achievement in adults. Acta Psychol. 140, 50–57. doi: 10.1016/J.ACTPSY.2012.02.008, PMID: 22445770

[ref63] R Core Team. (2017). R: A language and environment for statistical computing. R Foundation for Statistical Computing, Vienna, Austria.

[ref64] Reyna V. F. Brainerd C. J. (2007). The importance of mathematics in health and human judgment: numeracy, risk communication, and medical decision making. Learn. Individ. Differ. 17, 147–159. doi: 10.1016/j.lindif.2007.03.010

[ref65] Reynvoet B. Ribner A. D. Elliott L. Van Steenkiste M. Sasanguie D. Libertus M. E. (2021). Making sense of the relation between number sense and math. J. Numerical Cognition 7, 308–327. doi: 10.5964/jnc.6059

[ref66] Romano E. Babchishin L. Pagani L. S. Kohen D. (2010). School readiness and later achievement: replication and extension using a nationwide Canadian survey. Dev. Psychol. 46, 995–1007. doi: 10.1037/A0018880, PMID: 20822218

[ref67] Sasanguie D. Göbel S. M. Moll K. Smets K. Reynvoet B. (2013). Approximate number sense, symbolic number processing, or number-space mappings: what underlies mathematics achievement? J. Exp. Child Psychol. 114, 418–431. doi: 10.1016/j.jecp.2012.10.012 23270796

[ref68] Schneider M. Beeres K. Coban L. Merz S. Susan Schmidt S. Stricker J.. (2017). Associations of non-symbolic and symbolic numerical magnitude processing with mathematical competence: a meta-analysis. Dev. Sci. 20:e12372. doi: 10.1111/desc.12372, PMID: 26768176

[ref69] Smets K. Sasanguie D. Szücs D. Reynvoet B. (2015). The effect of different methods to construct non-symbolic stimuli in numerosity estimation and comparison. J. Cogn. Psychol. 27, 310–325. doi: 10.1080/20445911.2014.996568

[ref70] Sober E. (2002). Instrumentalism, parsimony, and the Akaike framework. Philos. Sci. 69, S112–S123. doi: 10.1086/341839

[ref71] Soltész F. Szűcs D. Szűcs L. (2010). Relationships between magnitude representation, counting and memory in 4- to 7-year-old children: a developmental study. Behav. Brain Funct. 6:13. doi: 10.1186/1744-9081-6-1, PMID: 20167066 PMC2833140

[ref72] Starr A. Libertus M. E. Brannon E. M. (2013). Number sense in infancy predicts mathematical abilities in childhood. Proceedings of the National Academy of Sciences 110, 18116–18120. doi: 10.1073/pnas.1302751110, PMID: 24145427 PMC3831472

[ref73] Szucs E. D. Nobes A. Devine A. Gabriel F. C. Gebuis T. (2013). Visual stimulus parameters seriously compromise the measurement of approximate number system acuity and comparative effects between adults and children. Front. Psychol. 4:444. doi: 10.3389/fpsyg.2013.00444, PMID: 23882245 PMC3715731

[ref74] ten Braak D. Lenes R. Purpura D. J. Schmitt S. A. Størksen I. (2022). Why do early mathematics skills predict later mathematics and reading achievement? The role of executive function. J. Exp. Child Psychol. 214:105306. doi: 10.1016/J.JECP.2021.105306, PMID: 34655996

[ref75] Vanbinst K. Ghesquiere P. De Smedt B. (2012). Representations and individual differences in children’ s arithmetic strategy use. Mind Brain Educ. 6, 129–136. doi: 10.1111/j.1751-228X.2012.01148.x

[ref76] Wilkey E. D. Shanley L. Sabb F. Ansari D. Cohen J. C. Men V.. (2021). Sharpening, focusing, and developing: a study of change in nonsymbolic number comparison skills and math achievement in 1st grade. Dev. Sci. 25:13194. doi: 10.1111/desc.13194, PMID: 34800342

[ref77] Zelazo P. D. (2006). The dimensional change card Sort (DCCS): a method of assessing executive function in children. Nat. Protoc. 1, 297–301. doi: 10.1038/nprot.2006.46, PMID: 17406248

